# Polyolefins, a Success Story

**DOI:** 10.3390/polym9060185

**Published:** 2017-05-24

**Authors:** Dominique W. Sauter, Mostafa Taoufik, Christophe Boisson

**Affiliations:** Université de Lyon, Univ Lyon 1, CPE Lyon, CNRS, UMR 5265, C2P2 (Chemistry, Catalysis, Polymers & Processes), Bat. 308F, 43 Bd du 11 Novembre 1918, 69616 Villeurbanne, France; dominique.sauter@gmail.com (D.W.S.); Mostafa.TAOUFIK@univ-lyon1.fr (M.T.)

**Keywords:** polyolefins, coordination catalysis, specialty polymers, high-performance resins, macromolecular engineering, tailor-made polymers, history of science

## Abstract

This paper reports the principal discoveries which have played a major role in the polyolefin field and have positioned polyolefins as the most produced plastics. The early development of polyolefins covering the production of LDPE (Low density polyethylene) at ICI (Imperial Chemical Industries) and the discovery of Phillips or Ziegler-Natta catalysts are highlighted in the first section. In the second part, the impact of the implementation of molecular catalysts on the research in polyolefins is discussed together with the most recent advances leading to high-performance tailor-made resins.

## 1. Introduction

Polyolefins, in particular polyethylene (PE) and isotactic polypropylene (*i*-PP), are widely used in our everyday life for an extremely wide range of applications; indeed, they account for more than 50% in weight of the produced polymers. More than 300 grades of commercially available polyolefins provide a wide range of mechanical properties [1]. With more than 178 million tons having been produced in 2015 [2,3], polyolefins remain at the top of the global production of synthetic polymers [4]. From the point of view of cost, PE and *i*-PP rank as the least expensive polymers to produce, and stand out because they are far less toxic compared to many other commodity plastics [5]. As discussed by Mülhaupt et al. [6], polyolefins meet the requirements of sustainable development and green polymer chemistry [7,8]. The development of solvent-free catalytic processes, the design of lightweight engineering plastics, the recyclability and the low carbon footprint of polyolefins are the main arguments for this statement. Polyethylene is the simplest polyolefin, its general formula is (–CH_2_–CH_2_–)_n_, and it is generally composed of a mixture of interconnected crystalline and amorphous parts which can exhibit short and long chain branching. Even if polyethylene seems to be an unsophisticated material in a first glance, it turns to be possible to tailor the polymer to include elaborate structures based on the organization of the three basic groups, methyl, methylene and methine. This also includes the preparation of blends.

**Scheme 1:**
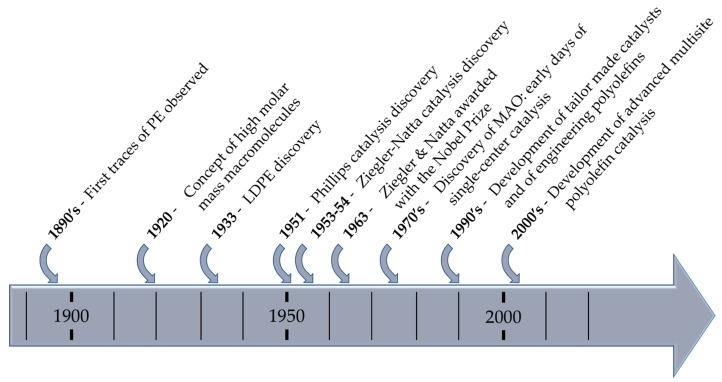
Major advances in polyolefins development.

Polyolefins are either produced by a free radical process (Low density polyethylene: LDPE) or using coordination catalysis (Low linear density polyethylene: LLDPE, high density polyethylene: HDPE, and *i*-PP). The present paper highlights the chronology of the development of the polyolefin production and the recent advances leading to performance polyolefins available today (Scheme 1).

## 2. Polyolefins, the Beginning of the Story: How Does It All Start?

Although the first trace of polyethylene was reported at the end of the 19th century as a side product formed during the thermal decomposition of diazomethane [9], it was only identified later as a polymer.

In the 1920s Hermann Staudinger set the basis for a new field of chemistry by introducing the concept of high molar mass macromolecules, and defining the polymerization process as linking together individual small monomer molecules by the formation of covalent bonds [10,11,12]. It was only a few years later, in 1933 at the Imperial Chemical Industries (ICI) site in Winnington (Northwich, UK) that Fawcett and Gibson discovered the overnight formation of a small amount (about 1 g) of an unexpected white powder while running some condensation experiments with highly pressurized ethylene and benzaldehyde [13,14]. When Fawcett and Gibson tried to repeat this experiment without benzaldehyde, the reactor exploded. It took two additional years to finally repeat the production of polyethylene, and yet again, the success of the test was obtained by chance. Indeed, while repeating this experiment, one of their autoclaves had a leak which allowed some traces of oxygen to contaminate the ethylene batch. At high temperature, the molecular oxygen decomposed to provide free radicals thereby enabling the formation of polyethylene [15]. In this free radical process, intra- and intermolecular chain transfer led to the formation of LDPE containing short and long chain branching. A process employing high temperature (200 to 300 °C) and high pressure (1000 to 4000 bars) was patented by ICI in 1937 [16], and the first factory production plant started operating in 1939 with a capacity of 100 ton/year [14]. Nowadays, LDPE is still widely produced with conditions close to those elaborated by Fawcett, Gibson and Perrin, and largely used in the food industry because it combines excellent mechanical properties and food friendly advantages (non-toxic and metal free materials).

Technological advances continued during the 1940s; several groups developed some efforts in order to find a catalytic process for the production of polyethylene. Mayo at U.S. Rubber Company and Fischer at BASF AG produced HDPE. The U.S. Rubber company did not believe in HDPE’s commercial potential [17], while BASF AG showed interest in Fischer’s catalysis work involving titanium tetrafluoride and aluminum powder [13]. However, during this troubled period, Fischer was asked to refocus his research in order to participate in the war effort by developing new types of fuel for the army. This considerably slowed down his advances in polyolefin catalysis, but finally his catalytic system was patented in 1953, ten years after its discovery [18].

It was in the early 1950s that two major breakthroughs happened in the field of catalytic polymerization of olefins. These discoveries enabled to run polymerization under milder temperature and pressure conditions than those previously used for LDPE production. The first major progress concerned the work done by Hogan and Banks at Phillips Petroleum Company, in Bartlesville, Oklahoma. Hogan and Banks research focused on the conversion of gaseous olefin to liquid oligomers, to produce fuel. For this purpose researchers at Phillips used to work with nickel oxides supported on silica or alumina [19,20,21]. In June 1951 while investigating the effect of a mixture of nickel oxides and chromium oxide compounds supported on silica/alumina, they unexpectedly plugged their reactor with solid HDPE [22]. They finely tuned their catalyst to the point where only chromium oxides were supported on silica. They found that they were able to polymerize ethylene with this new catalyst, and in October 1951 they also managed to polymerize propylene. They filed a patent application which was approved in 1953 [23,24,25]. Subsequently, they developed a commercial process by licensing this invention no later than 4 years after the discovery. Nowadays Phillips catalysts still represent 40 to 50% of the global production of HDPE, and the Phillips process remains quite popular because of its faculty to offer small amounts of long chain branching (LCB) via macromer insertion [26,27,28,29]. The existence of LCB offers major advantages in terms of mechanical properties, as well as easily controlled molding parameters [30].

The second major breakthrough occurring in the 1950s was the work of Ziegler at the Mülheim Max Planck Institute in Germany. Ziegler had been developing new types of organometallic compounds for a carbon-carbon bond formation purpose since the 1920s. At this time Ziegler’s favorite monomers were olefins, styrene and dienes. For example in 1928 he performed the polymerization of butadiene using alkyllithium as initiator [31]. However, the production of polyethylene using this method was limited because of the decomposition of lithium alkyls into lithium hydride and olefins. Ziegler found a way to prepare LiAlH_4_ as well as a direct pathway to produce alkylaluminum compounds directly from aluminum, hydrogen and olefins. He used those organometallic reagents to develop the so called “Aufbaureaktion” which enabled the preparation of 1-olefins and aliphatic alcohol (alfol synthesis), as well as high purity alumina via the oxidation of the alkylaluminum compounds. Due to fortuitous circumstances Ziegler realized that the presence of nickel changed the path of his “Aufbaureaktion” by favoring the chain termination of the reaction, leading to the formation of 1-butene. This phenomenon was called “the nickel effect”. They explained the presence of nickel in their Inox autoclave from either a previous hydrogenation reaction, or an aggressive cleaning of the reaction vessel. However, the identification of this phenomenon stood out as the beginning of an important metal screening performed by Ziegler’s students, in order to identify the behavior of different metal complexes while associated with some alkylaluminum compounds [32]. From August to October 1953 the association of triethylaluminum with several transition metal complexes, such as chromium, vanadium, manganese and platinum was evaluated. At that time, the most significant result was obtained by combining zirconium acetylacetonate with triethylaluminum, leading to a conversion above 90% of the introduced ethylene. Based on these results, Ziegler filed a patent application just 3 days after repeating the experiment. This patent application claimed “a process for the production of a high molar mass polyethylene useful as a plastic” involving the association of triethylaluminum with several complexes involving metals from group 4 (Ti, Zr, Hf), 5 (V, Nb, Ta) and 6 (Cr, Mo) [33]. In November 1953 titanium tetrachloride mixed with triethylaluminum was discovered to triple the productivity of the polymerization, and they were able to run the polymerization of ethylene at lower temperatures and with pressure of ethylene below 55 bars. This outcome prompted Ziegler to file a second patent in December 1953, claiming more gentle conditions than in the previous patent [34]. In March 1954, Natta successfully performed the stereospecific polymerization of propylene using a Ziegler catalyst (titanium trichloride/triethylaluminum). The isolated isotactic polypropylene was characterized by X-ray crystallography [35]. Natta patented the process for production of isotactic polypropylene [36,37,38,39]. Ziegler also filed a patent application for the production of *i*-PP, but it was 10 days too late [15]. Even if Natta was not the first to produce polypropylene, he nonetheless introduced the concept of stereoregularity and participated actively in the development of the polyolefin field [40]. In 1957 the first plant producing isotactic polypropylene using Ziegler-Natta technology opened in Ferrara, Italy [41]. In the 1960s, Ziegler-Natta catalysts were extensively studied in order to maximize the activity and to improve the stereospecificity of propylene polymerization. In particular, the catalysts were supported on various surfaces, especially on MgCl_2_. The impact of the work in the field of olefin polymerization done by Ziegler and Natta was rewarded with the Noble Prize in Chemistry in 1963 [42]. Because of the importance of Ziegler’s invention, many companies tried to invalidate the patent filed by Ziegler in 1953, arguing that Fischer’s work at BASF described suitable conditions to form Ziegler’s catalysts. However a US court decision of 1967 proved them wrong and maintained Ziegler’s protection [43].

## 3. Single-Center Technology

In the mid. 1970s, another “incident” led to the discovery of a chemical species which changed the world of polymerization catalysis [44]. Indeed, at the Institute of Technical and Macromolecular Chemistry at the University of Hamburg, Sinn and Kaminsky were investigating side reactions occurring while using Ziegler’s catalysts [45,46]. They monitored their experiments by NMR, and in order to facilitate the interpretation of the NMR, the reaction between trimethylaluminum and bis(cyclopentadienyl)titanium dimethyl was chosen as model. In the presence of ethylene, and after five hours of reaction, moisture from the air passed thought the plastic lid causing the unexpected formation of PE. A following experiment at larger scale clearly confirmed the presence of polyethylene. Researchers suspected an unidentified species to be responsible of this phenomenon, so they tried to screen multiple possibilities such as a side reaction of AlMe_3_ with chlorides, oxygen or water. After eliminating the two first hypothesis, it turned out that water reacting with trimethylaluminum formed a new compound which enhanced catalytic activity when associated with a titanocene [47]. This new compound called methylaluminoxane, referred as MAO, is commonly represented as (–Al–O(–Me)–)_n_. The overall composition of MAO includes some free AlMe_3_ and it is generally admitted to say that MAO is a cluster of aluminum, oxygen and methyl groups with the general formulae [–(CH_3_)_x_AlO_(3-x)/2_^−^] [48]. The presence of free AlMe_3_ in commercial MAO plays a crucial role on the catalytic activity and chain transfer to aluminum via the formation of dormant heterobimetallic species [49].

Recently, computational exploration has allowed researchers to outline several energetically favored MAO structures, and deeper investigations enabled them to demonstration that MAO dissociation led to a [AlMe_2_]^+^ cation, which was suspected to play a major role in the process of metallocene activation [50,51]. The discovery of MAO was the starting point for the wider development of metallocene catalysts [52]. Jordan et al. identified the active site responsible for olefin polymerization while using an activated metallocene as a cationic species [53,54]. Beyond group 4 metallocenes, it was shown that MAO can be used to activate a wide range of molecular complexes of early and late transition metals, leading to a new generation of catalysts with tailor-made properties. These catalysts, based on well-defined precursors, are also named “single-center catalysts” [55]. As an example, in 1991, Exxon-Mobil launched its Exxpol™ Technology for production of polyolefins using a metallocene catalyst [56]. In addition to the gain of productivity enabled by MAO activation of the metallocene, these catalysts were also used to prepare stereoregular polyolefins [55,57,58] such as isotactic polypropylene (*i*-PP) [59,60], syndiotactic polypropylene (*s*-PP) [61], hemi-isotactic polypropylene (*hi*-PP) [62] and stereoblock polypropylene [63,64,65,66]. The relation between the symmetry of the metallocene center and the polypropylene microstructure can be established using Ewen’s stereocontrol rules [67]. These tailor-made catalysts for propylene polymerization constituted the second breakthrough generated by MAO discovery. Hemi-metallocenes were also developed in order to fit to specific need in terms of polyolefin properties [68]. In particular *ansa*-cyclopentadienyl-amido titanium complexes [69,70,71] were designed and implemented for ethylene polymerization, and were referred as constrained geometry catalysts (CGC) [72,73,74,75]. Dow’s INSITE™ CGC catalyst technology was launched in 1992 [56]. CGC catalysts enabled better insertion of co-monomer (especially α-olefins) than regular metallocenes, because of a less hindered coordination sphere, coupled to a Cp(centroid)-Ti-N with a smaller bite angle [76]. This class of complexes offered enhanced thermal stability, allowing them to be used at high temperature in the Dowlex™ solution process [77]. A vast range of non-metallocene molecular catalysts were developed during the last three decades leading to an outstanding variety of accessible polyolefin materials [68,78,79,80,81,82].

Subsequently, molecular activators able to activate metallocene (or post metallocene) complexes under a cationic form were discovered [83,84]. For example the tris(pentafluorophenyl)borane which was synthesized a decade before the discovery of MAO [85,86], was independently identified in the early 1990 by Marks [87,88] and Ewen [89,90] as an activator for group 4 metallocene complexes. The activation of metallocene complexes with tetraperfluoroarylborate salt ([Ph_3_C]^+^[B(C_6_F_5_)_4_]^−^ [91,92] or [HNRR’_2_]^+^[B(C_6_F_5_)_4_]^−^ [93,94,95]) led to the formation of dissociated ion pair species which featured increased activity in olefin polymerization. It is worth mentioning the role of dissociation of the catalyst ion pair (inner and outer ion pairs) in polymerization [96,97].

As in the case of classical Ziegler-Natta catalyst, the presence of main group organometallic species (AlR_3_, MgR_2_) or ZnEt_2_ in polymerization medium led to chain transfer to metal. Interestingly a fast and reversible chain transfer provided a unique method for controlled coordination polymerization of olefins [98,99,100,101].

## 4. Developments in Polyolefins Driven by Molecular Catalysts

For two decades since MAO metallocene activation, many improvements in single-site technology were realized as described above. Besides the activity gain and the access to unique stereoregular types of polypropylene (*s*-PP; *hi*-PP or stereoblock-PP), molecular catalyst evolution allowed the development of specialty polyolefins. For instance, ethylene/CO copolymers (polyketones—Carilon™) [102,103,104] and cycloolefin copolymers (COC) [105] were marketed by respectively Shell chemicals (discontinued in 2000) and Ticona. Dow launched new plastomers (AFFINITY™), polyolefin elastomers (ENGAGE™) and performance EPDM (NORDEL™) based on their INSITE™ technology [56]. The same company discovered non metallocene hafnium catalysts (pyridyl-amido hafnium catalyst) for the stereospecific polymerization of propylene at high temperature [81]. Propylene/ethylene copolymers were produced in a solution process and commercialized as plastomers and elastomers (VERSIFY™).

Bis(phenoxy-imine) group 4 catalysts (FI catalysts) are another major class of molecular catalysts introduced by Mitsui Chemicals. These catalysts showed high efficiency for olefin polymerization and were also very versatile [82,106,107]. Changing the substituent of the phenoxy-imine ligands, the metal (Ti and Zr) or the activator can lead to the production of a wide range of polyolefins, including ultrahigh molecular weight polyethylene, vinyl-terminated polyethylene that was turned to functional polyethylene, and isotactic or syndiotactic polypropylene. Note that Dow Chemicals used a FI catalyst in association with their pyridyl-amido hafnium catalyst and ZnEt_2_ to produce multiblock copolymers (see below). Intense research efforts at DSM (acquired by Lanxess in 2011, now Arlanxeo) led to the development of cyclopentadienyl-amidinato titanium catalysts for the production of high-performance EPDM (Keltan Ace™) [68]. The copolymerization of olefins with conjugated dienes was a challenge since these two classes of monomers polymerized via different mechanisms. Neodymium metallocene catalysts showed unprecedented copolymerization of ethylene with butadiene leading to a new class of elastomers [108,109,110]. Recently, Perrin et al. used computational investigation to demonstrate that a heterobimetallic and a monomeric intermediate were formed after insertion of ethylene and butadiene respectively [111]. More generally, progress in computational investigation using DFT as modeling method led to accurate prediction of the behavior of olefin polymerization catalysts [112].

For a long time, conventional multi-site catalysts (Ziegler-Natta) were opposed to molecular catalysts namely because of the lack of control in terms of chemical composition distribution leading to branching in the lower molar mass fraction of LLDPE. Importantly, researchers turned this paradigm around by switching from conventional multi-site to advanced multi-site catalysts via ingenious combinations of catalysts enabling the production of polyolefins with tailor-made properties. We can define two classes of multi-site catalysts (i) hybrid catalysts which combine individual effects of each component and (ii) concurrent tandem catalysts which lie on cooperative effect between each component. Hybrid catalysts were developed to produce high-performance bimodal resins where branching were preferentially located in the high molar mass fraction. These resins represent a new class of high-performance polyolefins. Single-center technology provides polyolefins with a low dispersity (*Ð* around 2), but in many instances it is essential to offer a resin which presents a broader molecular weight distribution in order to limit the shear forces involved during extrusion. In addition, mechanical properties can be largely improved by introducing branching in the high molar mass fraction. It is the concept of reverse comonomer distribution. This topic was comprehensively reviewed by Friederichs et al. [113] and Mülhaupt et al. [6]. It is worth pointing out that bimodal resins can not only be produced using combination of catalysts in a single reactor [114], but also by an engineering approach, in a cascade of (continuous) reactors [115,116,117], or multizone reactors [118,119]. The preparation of bimodal resins using hybrid catalysts has been evaluated since the early times of single site catalysts. Indeed Ewen and Welborn investigated the combination of different metallocenes in solution process [120,121]. In the case of particle-forming processes (slurry and gas phase), tremendous advances were achieved by combining supported catalysts (Ziegler-Natta catalysts, Phillips catalysts, supported molecular catalysts). The Prodigy BMC catalyst, commercialized by Univation Technologies, constitutes one of the better known classes of hybrid catalysts obtained by the combination of two metallocene complexes [122,123,124]. In 2016, Mülhaupt reported the synthesis of a tri-site catalyst supported on clay. This catalyst involved two different types of Cr based centers (CrBIP and CrQCp) associated to either a Zr based center (ZrCp) or to a Fe based center (FeBIP). The polymerization of ethylene with this tri-site catalyst afforded a tri-modal resin presenting extraordinary enhanced properties [125]. Independently of the techniques used to produce mutlimodal resins, this challenge led to a major competition between polyolefin producers and many advances were reported, in particular by Univation Technologies, Ineos, Chevron Phillips Chemical and LyondellBasell [6,113]. Multimodal resins produced in this manner offer significant gains in terms of processability, stiffness and crack resistance.

Tandem catalysts were developed for the preparation of branched polyolefins with ethylene using the combination of oligomerization and polymerization catalysts [126,127,128,129,130,131]. Interestingly, the combination of oligomerization catalysts leading to macromers with a CGC catalysts permitted the production of branch-block thermoplastic elastomers [132]. Recently, binuclear catalysts based on an olefin polymerization catalyst part (typically a Ti based CGC complex) linked to an ethylene trimerization catalyst part (typically a SNS-Cr based complex) were successfully synthesized by Marks et al. From ethylene feed and activation of this cooperative Ti-SNS-Cr catalysts by MAO, the resulting resin displayed only butyl branches (LLDPE) [133,134].

In a different manner, synergetic effects were also disclosed leading to unique performance polyolefins. Researchers at the Dow Chemical Company introduced the concept of chain shuttling combining two catalysts (a FI zirconium and a pyridyl-amido hafnium catalyst) with different comonomer responses and a chain shuttling agent (ZnEt_2_) transferring the growing chain from one metal to the other one. Olefin block copolymers (OBCs) bearing soft and hard polyolefin sequences were marketed (INFUSE™) and led to a new area of research [135,136,137,138].

Most of single-site catalyst’s development were first optimized for homogeneous catalysis, for example Dow’s INSITE™ technology which involved CGC catalysts was developed initially for solution process at high temperatures [56,139,140,141,142,143]. Today, solution processes, such as “Compact Process” from Borealis, “Dowlex” from Dow Chemicals, or “Sclairtech” from Nova Chemicals enable the production of a wide variety of commodity or specialty polyolefins [144]. Nevertheless, solution processes are energy consuming (high temperature) and require large amount of solvent. Most of the major industrial processes for olefin polymerization are heterogeneous (slurry and gas phase processes). In these processes, the polymer crystallizes during the polymerization and it is very important to control the polymer particle morphology. This requires the use of solid catalysts which can fragment and lead to spherical polyolefin particles. Thus, the use of molecular catalysts in particle forming processes requires the immobilization of the catalyst on a support such as silica or MgCl_2_. This step is far from being trivial and requires many efforts in order to combine a high efficiency, with the production of spherical particles and resins with expected properties, without a significant generation of heat, nor active species leaching. This major research area was remarkably reviewed in a comprehensive book edited by Severn and Chadwick [1] and in review articles [144,145].

## 5. Conclusions

To conclude, even though Polyolefins were discovered and identified many years ago, this research field is constantly evolving. Multiple side-events led to major breakthroughs, enabling us to finely adjust polyolefins’ properties. More than 60 years after the first catalytic processes, the fields of polyolefin catalysis remains a very active field of research, which has been greatly transformed over time, passing from intrinsically multisite catalysts, to new advanced multi-site catalysts, leading year after year to more advanced materials. In the future, polyolefins will keep its leading role in the plastic industry and will continue to offer a wide pallet of solutions worldwide. Finally, we could paraphrase Vincenzo Busico highlighting 10 years ago the emergence of the concept of degenerative chain transfer, and in particular chain shuttling polymerization for the production of olefin block copolymers, the “success” story continues [146].

## References

[1] Severn J.R., Chadwick J.C. (2008). Tailor-Made Polymers.

[2] AlMa’adeed M.A.-A., Krupa I., AlMa’adeed M.A.-A., Krupa I. (2016). Introduction. Polyolefin Compounds and Materials.

[3] Hutley T.J., Ouederni M., AlMa’adeed M.A.-A., Krupa I. (2016). Polyolefins—The History and Economic Impact. Polyolefin Compounds and Materials.

[4] Stalzer M.M., Delferro M., Marks T.J. (2014). Supported Single-Site Organometallic Catalysts for the Synthesis of High-Performance Polyolefins. Catal. Lett..

[5] Tabone M.D., Cregg J.J., Beckman E.J., Landis A.E. (2010). Sustainability Metrics: Life Cycle Assessment and Green Design in Polymers. Environ. Sci. Technol..

[6] Stürzel M., Mihan S., Mülhaupt R. (2016). From Multisite Polymerization Catalysis to Sustainable Materials and All-Polyolefin Composites. Chem. Rev..

[7] Anastas P.T., Warner J.C. (1998). Green Chemistry: Theory and Practice.

[8] Mülhaupt R. (2013). Green Polymer Chemistry and Bio-based Plastics: Dreams and Reality. Macromol. Chem. Phys..

[9] Pechmann H.V. (1898). Ueber Diazomethan und Nitrosoacylamine. Berichte der Deutschen Chemischen Gesellschaft.

[10] Staudinger H. (1920). Über Polymerisation. Berichte der Deutschen Chemischen Gesellschaft B.

[11] Staudinger H., Fritschi J. (1922). Über Isopren und Kautschuk. 5. Mitteilung. Über die Hydrierung des Kautschuks und über seine Konstitution. Helv. Chim. Acta.

[12] Staudinger H. (1924). Über die Konstitution des Kautschuks (6. Mitteilung). Berichte der Deutschen Chemischen Gesellschaft B.

[13] Seymour R.B., Cheng T. (1985). History of Polyolefins.

[14] Utracki L.A. (2000). Polymer Blends.

[15] Seymour R.B., Cheng T. (1987). Advances in Polyolefins.

[16] Fawcett E.W., Gibson R.O. (1937). Improvements in or Relating to the Polymerisation of Ethylene. Patent.

[17] McMillan F.M. (1979). The Chain Straighteners: Fruitful Innovation; the Discovery of Linear and Stereoregular Synthetic Polymers.

[18] Fischer M.D. (1953). Verfahren zur Herstellung von festen Polymerisaten aus AEthylen oder aethylenreichenGasen. Patent.

[19] Bailey G.C., Reid J.A. (1945). Catalytic Polymerization of Olefins. U.S. Patent.

[20] Bailey G.C., Reid J.A. (1952). SiO_2_-Al_2_O_3_-NiO Catalyst and Its Preparation. U.S. Patent.

[21] Bailey G.C., Reid J.A. (1952). Catalytic Polymerization of Olefins. U.S. Patent.

[22] Sailors H.R., Hogan J.P. (1981). History of Polyolefins. J. Macromol. Sci. Part. Chem..

[23] Hogan J.P., Banks R.L. (1958). Polymers and Production Thereof. U.S. Patent.

[24] Hogan J.P., Banks R.L. (1957). Improvements in or Relating to Polymerization of Olefins. Patent.

[25] Hogan J.P., Banks R.L. (1957). Perfectionnements aux Polymères et à Leur Production par Action Catalytique. Patent.

[26] Hogan J.P. (1970). Ethylene polymerization catalysis over chromium oxide. J. Polym. Sci..

[27] Hogan J.P. (1983). Ch.6—Catalysis of the Phillips Petroleum Company Polyethylene Process. Applied Industrial Catalysis.

[28] McDaniel M.P. (1985). Supported Chromium Catalysts for Ethylene Polymerization. Advances in Catalysis.

[29] McDaniel M.P., Rohlfing D.C., Benham E.A. (2003). Long Chain Branching in Polyethylene from the Phillips Chromium Catalyst. Polym. React. Eng..

[30] Janzen J., Colby R.H. (1999). Diagnosing long-chain branching in polyethylenes. J. Mol. Struct..

[31] Ziegler K., Bähr K. (1928). Über den vermutlichen Mechanismus der Polymerisationen durch Alkalimetalle (Vorläufige Mitteilung). Berichte der Deutschen Chemischen Gesellschaft B.

[32] Ziegler K. (1968). A Forty Years’ Stroll through the Realms of Organometallic Chemistry. Advances in Organometallic Chemistry.

[33] Ziegler K., Heinz B., Erhard H., Heinz M. (1960). High Molecular Polyethylenes. Patent.

[34] Ziegler K., Breil H., Martin H. (1957). High molecular polyethylenes. Patent.

[35] Natta G., Pino P., Corradini P., Danusso F., Mantica E., Mazzanti G., Moraglio G. (1955). Crystalline high polymers of α-olefins. J. Am. Chem. Soc..

[36] Natta G., Pino P., Mazzanti G. (1966). Prevailingly to Substantially Atactic Crude Polymers and Methods for Producing the Same. U.S. Patent.

[37] Natta G., Pasquon I. (1966). Process for Polymerizing Unsaturated Hydrocarbons to Crystalline Polymers of Regulated Molecular Weight. U.S. Patent.

[38] Natta G., Crespi G. (1965). Block Polymers of Alpha-Olefines, Processes for Producing the Same, and Mixtures Thereof with Isotactic Polyolefines. U.S. Patent.

[39] Natta G., Pino P., Mazzanti G. (1963). Isotactic Polypropylene. U.S. Patent.

[40] Corradini P. (1995). The impact of the discovery of stereoregular polymers in macromolecular science. Macromol. Symp..

[41] Galli P. (1995). Forty years of industrial developments in the field of isotactic polyolefins. Macromol. Symp..

[42] The Nobel Prize in Chemistry 1963. http://www.nobelprize.org/nobel_prizes/chemistry/laureates/1963/.

[43] Martin H. (2007). Polymers, Patents, Profits: A Classic Case Study for Patent Infighting.

[44] Kaminsky W. (2012). Discovery of Methylaluminoxane as Cocatalyst for Olefin Polymerization. Macromolecules.

[45] Sinn H., Hinck H., Bandermann F., Grützmacher H.F. (1968). Bildung von Poly(methyl-methylen-aluminium). Angew. Chem..

[46] Kaminsky W., Vollmer H.-J., Heins E., Sinn H. (1974). Die Bildung von Dimetalloalkylenen, eine unvermeidliche Nebenreaktion homogener ZIiegler-Katalysatoren. Makromol. Chem..

[47] Andresen A., Cordes H.-G., Herwig J., Kaminsky W., Merck A., Mottweiler R., Pein J., Sinn H., Vollmer H.-J. (1976). Halogen-Free Soluble Ziegler Catalysts for the Polymerization of Ethylene. Control of Molecular Weight by Choice of Temperature. Angew. Chem. Int. Ed. Engl..

[48] Imhoff D.W., Simeral L.S., Sangokoya S.A., Peel J.H. (1998). Characterization of Methylaluminoxanes and Determination of Trimethylaluminum Using Proton NMR. Organometallics.

[49] Ehm C., Cipullo R., Budzelaar P.H.M., Busico V. (2016). Role(s) of TMA in polymerization. Dalton Trans..

[50] Ghiotto F., Pateraki C., Tanskanen J., Severn J.R., Luehmann N., Kusmin A., Stellbrink J., Linnolahti M., Bochmann M. (2013). Probing the Structure of Methylalumoxane (MAO) by a Combined Chemical, Spectroscopic, Neutron Scattering, and Computational Approach. Organometallics.

[51] Hirvi J.T., Bochmann M., Severn J.R., Linnolahti M. (2014). Formation of Octameric Methylaluminoxanes by Hydrolysis of Trimethylaluminum and the Mechanisms of Catalyst Activation in Single-Site α-Olefin Polymerization Catalysis. ChemPhysChem.

[52] Kaminsky W. (2004). The discovery of metallocene catalysts and their present state of the art. J. Polym. Sci. Part. A Polym. Chem..

[53] Jordan R.F., Dasher W.E., Echols S.F. (1986). Reactive cationic dicyclopentadienyl zirconium(IV) complexes. J. Am. Chem. Soc..

[54] Jordan R.F., Bajgur C.S., Willett R., Scott B. (1986). Ethylene polymerization by a cationic dicyclopentadienyl zirconium(IV) alkyl complex. J. Am. Chem. Soc..

[55] Resconi L., Cavallo L., Fait A., Piemontesi F. (2000). Selectivity in Propene Polymerization with Metallocene Catalysts. Chem. Rev..

[56] Chum P.S., Swogger K.W. (2008). Olefin polymer technologies—History and recent progress at The Dow Chemical Company. Prog. Polym. Sci..

[57] Corradini P., Guerra G., Cavallo L. (2004). Do New Century Catalysts Unravel the Mechanism of Stereocontrol of Old Ziegler—Natta Catalysts?. Acc. Chem. Res..

[58] Busico V., Cipullo R. (2001). Microstructure of polypropylene. Prog. Polym. Sci..

[59] Ewen J.A. (1984). Mechanisms of stereochemical control in propylene polymerizations with soluble Group 4B metallocene/methylalumoxane catalysts. J. Am. Chem. Soc..

[60] Kaminsky W., Külper K., Brintzinger H.H., Wild F.R.W.P. (1985). Polymerisation von Propen und Buten mit einem chiralen Zirconocen und Methylaluminoxan als Cokatalysator. Angew. Chem..

[61] Ewen J.A., Jones R.L., Razavi A., Ferrara J.D. (1988). Syndiospecific propylene polymerizations with Group IVB metallocenes. J. Am. Chem. Soc..

[62] Ewen J.A., Elder M.J., Jones R.L., Haspeslagh L., Atwood J.L., Bott S.G., Robinson K. (1991). Metallocene/polypropylene structural relationships: Implications on polymerization and stereochemical control mechanisms. Makromol. Chem. Macromol. Symp..

[63] Coates G.W., Waymouth R.M. (1995). Oscillating Stereocontrol: A Strategy for the Synthesis of Thermoplastic Elastomeric Polypropylene. Science.

[64] Wild F.R.W.P., Zsolnai L., Huttner G., Brintzinger H.H. (1982). Ansa-Metallocene Derivatives: IV. Synthesis and molecular structures of chiral ansa-titanocene derivatives with bridged tetrahydroindenyl ligands. J. Organomet. Chem..

[65] Schnutenhaus H., Brintzinger H.H. (1979). 1,1’-Trimethylenebis(η^5−3^-tert-butylcyclopentadienyl)-titanium(IV)Dichloride, a Chiral ansa-Titanocene Derivative. Angew. Chem. Int. Ed. Engl..

[66] Brintzinger H.H., Fischer D., Mülhaupt R., Rieger B., Waymouth R.M. (1995). Stereospecific Olefin Polymerization with Chiral Metallocene Catalysts. Angew. Chem. Int. Ed. Engl..

[67] Ewen J.A. (1998). Symmetry rules and reaction mechanisms of Ziegler–Natta catalysts1. J. Mol. Catal. Chem..

[68] Baier M.C., Zuideveld M.A., Mecking S. (2014). Post-Metallocenes in the Industrial Production of Polyolefins. Angew. Chem. Int. Ed..

[69] Shapiro P.J., Bunel E., Schaefer W.P., Bercaw J.E. (1990). Scandium complex [{(η^5^-C_5_Me_4_)Me_2_Si(η^1^-NCMe_3_)}(PMe_3_)ScH]_2_: A unique example of a single-component α-olefin polymerization catalyst. Organometallics.

[70] Gielens E.E., Tiesnitsch J.Y., Hessen B., Teuben J.H. (1998). Titanium Hydrocarbyl Complexes with a Linked Cyclopentadienyl—Alkoxide Ancillary Ligand; Participation of the Ligand in an Unusual Activation of a (Trimethylsilyl)methyl Group. Organometallics.

[71] Piers W.E., Shapiro P.J., Bunel E.E., Bercaw J.E. (1990). Coping with Extreme Lewis Acidity: Strategies for the Synthesis of Stable, Mononuclear Organometallic Derivatives of Scandium. Synlett.

[72] Chen Y.-X., Fu P.-F., Stern C.L., Marks T.J. (1997). A Novel Phenolate “Constrained Geometry” Catalyst System. Efficient Synthesis, Structural Characterization, and α-Olefin Polymerization Catalysis. Organometallics.

[73] Cano J., Kunz K. (2007). How to synthesize a constrained geometry catalyst (CGC)—A survey. J. Organomet. Chem..

[74] McKnight A.L., Waymouth R.M. (1998). Group 4 ansa-Cyclopentadienyl-Amido Catalysts for Olefin Polymerization. Chem. Rev..

[75] Babinec Susan S., Blanchard M., Guest M.J., Walther B., Chaudhary B.I., Barry R.P. (2000). Compositions of Interpolymers of Alpha-Olefin Monomers with One or More Vinyl or Vinylidene Aromatic Monomers. European Patent.

[76] Braunschweig H., Breitling F.M. (2006). Constrained geometry complexes—Synthesis and applications. Coord. Chem. Rev..

[77] Soares J.B.P., McKenna T.F.L. (2013). Polyolefin Reaction Engineering.

[78] Gibson V.C., Spitzmesser S.K. (2003). Advances in Non-Metallocene Olefin Polymerization Catalysis. Chem. Rev..

[79] Zuccaccia C., Macchioni A., Busico V., Cipullo R., Talarico G., Alfano F., Boone H.W., Frazier K.A., Hustad P.D., Stevens J.C. (2008). Intra- and Intermolecular NMR Studies on the Activation of Arylcyclometallated Hafnium Pyridyl-Amido Olefin Polymerization Precatalysts. J. Am. Chem. Soc..

[80] Heurtefeu B., Vaultier F., Leino R., Boisson C., Cramail H. (2014). Single-Site Catalysts. Encyclopedia of Polymer Science and Technology.

[81] Boussie T.R., Diamond G.M., Goh C., Hall K.A., LaPointe A.M., Leclerc M.K., Murphy V., Shoemaker J.A.W., Turner H., Rosen R.K. (2006). Nonconventional Catalysts for Isotactic Propene Polymerization in Solution Developed by Using High-Throughput-Screening Technologies. Angew. Chem. Int. Ed..

[82] Makio H., Kashiwa N., Fujita T. (2002). FI Catalysts: A New Family of High Performance Catalysts for Olefin Polymerization. Adv. Synth. Catal..

[83] Chen E.Y.-X., Marks T.J. (2000). Cocatalysts for Metal-Catalyzed Olefin Polymerization: Activators, Activation Processes, and Structure-Activity Relationships. Chem. Rev..

[84] Bochmann M. (2010). The Chemistry of Catalyst Activation: The Case of Group 4 Polymerization Catalysts. Organometallics.

[85] Massey A.G., Park A.J. (1964). Perfluorophenyl derivatives of the elements: I. Tris(pentafluorophenyl)boron. J. Organomet. Chem..

[86] Massey A.G., Park A.J. (1966). Perfluorophenyl derivatives of the elements: VII. further studies on tris(pentafluorophenyl)boron. J. Organomet. Chem..

[87] Yang X., Stern C.L., Marks T.J. (1991). Cation-like homogeneous olefin polymerization catalysts based upon zirconocene alkyls and tris(pentafluorophenyl)borane. J. Am. Chem. Soc..

[88] Yang X., Stern C.L., Marks T.J. (1994). Cationic Zirconocene Olefin Polymerization Catalysts Based on the Organo-Lewis Acid Tris(pentafluorophenyl)borane. A Synthetic, Structural, Solution Dynamic, and Polymerization Catalytic Study. J. Am. Chem. Soc..

[89] Ewen J.A., Elder M.J. (1996). Metallocene Catalysts with Lewis Acids and Aluminum Alkyls. Patent.

[90] Ewen J.A., Elder M.J. (1996). Metallocene Catalysts with Lewis Acids and Aluminum Alkyls. U.S. Patent.

[91] Chien J.C.W., Tsai W.M., Rausch M.D. (1991). Isospecific polymerization of propylene catalyzed by *rac*-ethylenebis(indenyl)methylzirconium cation. J. Am. Chem. Soc..

[92] Ewen J.A., Elder M.J. (1995). Preparation of metallocene catalysts for polymerization of olefins. Patent.

[93] Turner H.W. (1988). Catalysts, method of preparing these catalysts and method of using said catalysts. Patent.

[94] Hlatky G., Upton D., Turner H. (1991). Supported Ionic Metallocene Catalysts for Olefin Polymerization. Patent.

[95] Yang X., Stern C., Marks T.J. (1991). Models for organometallic molecule-support complexes. Very large counterion modulation of cationic actinide alkyl reactivity. Organometallics.

[96] Macchioni A. (2005). Ion Pairing in Transition-Metal Organometallic Chemistry. Chem. Rev..

[97] Ciancaleoni G., Fraldi N., Budzelaar P.H.M., Busico V., Cipullo R., Macchioni A. (2010). Structure—Activity Relationship in Olefin Polymerization Catalysis: Is Entropy the Key?. J. Am. Chem. Soc..

[98] Valente A., Mortreux A., Visseaux M., Zinck P. (2013). Coordinative Chain Transfer Polymerization. Chem. Rev..

[99] Sita L.R. (2009). Ex Uno Plures (“Out of One, Many”): New Paradigms for Expanding the Range of Polyolefins through Reversible Group Transfers. Angew. Chem. Int. Ed..

[100] Kempe R. (2007). How to Polymerize Ethylene in a Highly Controlled Fashion?. Chem. Eur. J..

[101] D’Agosto F., Boisson C. (2010). A RAFT Analogue Olefin Polymerization Technique Using Coordination Chemistry. Aust. J. Chem..

[102] Drent E. (1984). Process for the Preparation of Polyketones. Patent.

[103] Drent E., Van Broekhoven J.A.M., Doyle M.J. (1991). Efficient palladium catalysts for the copolymerization of carbon monoxide with olefins to produce perfectly alternating polyketones. J. Organomet. Chem..

[104] Drent E., Mul W.P., Smaardijk A.A. (2002). Polyketones. Encyclopedia of Polymer Science and Technology.

[105] Goodall B.L., Buschow K.H.J., Cahn R.W., Flemings M.C., Ilschner B., Kramer E.J., Mahajan S., Veyssière P. (2001). Cycloaliphatic Polymers. Encyclopedia of Materials: Science and Technology.

[106] Makio H., Fujita T. (2009). Development and Application of FI Catalysts for Olefin Polymerization: Unique Catalysis and Distinctive Polymer Formation. Acc. Chem. Res..

[107] Mitani M., Saito J., Ishii S., Nakayama Y., Makio H., Matsukawa N., Matsui S., Mohri J., Furuyama R., Terao H. (2004). FI Catalysts: New olefin polymerization catalysts for the creation of value-added polymers. Chem. Rec..

[108] Thuilliez J., Ricard L., Nief F., Boisson F., Boisson C. (2009). Ansa-Bis(fluorenyl)neodymium Catalysts for Cyclocopolymerization of Ethylene with Butadiene. Macromolecules.

[109] Llauro M.F., Monnet C., Barbotin F., Monteil V., Spitz R., Boisson C. (2001). Investigation of Ethylene/Butadiene Copolymers Microstructure by ^1^H and ^13^C NMR. Macromolecules.

[110] Belaid I., Monteil V., Boisson C., Hoff R., Tthers R. (2017). Handbook of Transition Metal Polymerization Catalysts.

[111] Ribeiro R., Ruivo R., Nsiri H., Norsic S., D’Agosto F., Perrin L., Boisson C. (2016). Deciphering the Mechanism of Coordinative Chain Transfer Polymerization of Ethylene Using Neodymocene Catalysts and Dialkylmagnesium. ACS Catal..

[112] Zaccaria F., Ehm C., Budzelaar P.H.M., Busico V. (2017). Accurate Prediction of Copolymerization Statistics in Molecular Olefin Polymerization Catalysis: The Role of Entropic, Electronic, and Steric Effects in Catalyst Comonomer Affinity. ACS Catal..

[113] Friederichs N., Ghalit N., Xu W., Severn J.R., Chadwick J.C. (2008). Chptr8—Supported Multicomponent Single-Site α-Olefin Polymerization Catalysts. Tailor-Made Polymers.

[114] Liu H.-T., Davey C.R., Shirodkar P.P. (2003). Bimodal polyethylene products from UNIPOL™ single gas phase reactor using engineered catalysts. Macromol. Symp..

[115] Ruff M., Paulik C. (2012). Controlling Polyolefin Properties by In-Reactor Blending, 1–Polymerization Process, Precise Kinetics, and Molecular Properties of UHMW-PE Polymers. Macromol. React. Eng..

[116] Ruff M., Paulik C. (2013). Controlling Polyolefin Properties by In-Reactor Blending: 2. Particle Design. Macromol. React. Eng..

[117] Ruff M., Lang R.W., Paulik C. (2013). Controlling Polyolefin Properties by In-Reactor Blending: 3. Mechanical Properties. Macromol. React. Eng..

[118] Mei G., Herben P., Cagnani C., Mazzucco A. (2006). The Spherizone Process: A New PP Manufacturing Platform. Macromol. Symp..

[119] Dorini M., Mei G., Cavani F., Centi G., Perathoner S., Trifiró F. (2009). Basell Spherizone Technology. Sustainable Industrial Chemistry.

[120] Ewen J.A., Welborn J. (1985). Process and Catalyst for Producing Polyethylene Having a Broad Molecular Weight Distribution. U.S. Patent.

[121] Ewen J.A., Welborn J. (1990). Process and Catalyst for Producing Reactor Blend Polyolefins. U.S. Patent.

[122] Lue C.-T., Crowther D.J. (2002). Mixed Catalysts for Use in a Polymerization Process. U.S. Patent.

[123] Vaughan G.A., Szul J.F., Mckee M.G., Farley J.M., Lue C.-T., Kao S.-C. (2006). Mixed Metallocene Catalyst Systems Containing a Poor Comonomer Incorporator and a Good Comonomer Incorporator. U.S. Patent.

[124] Liu H.-T., Mure C.R. (2013). Polyethylene Compositions. U.S. Patent.

[125] Stürzel M., Hees T., Enders M., Thomann Y., Blattmann H., Mülhaupt R. (2016). Nanostructured Polyethylene Reactor Blends with Tailored Trimodal Molar Mass Distributions as Melt-Processable All-Polymer Composites. Macromolecules.

[126] Denger C., Haase U., Fink G. (1991). Simultaneous oligomerization and polymerization of ethylene. Makromol. Chem. Rapid Commun..

[127] Ye Z., AlObaidi F., Zhu S. (2004). A Tandem Catalytic System for the Synthesis of Ethylene–Hex-1-ene Copolymers from Ethylene Stock. Macromol. Rapid Commun..

[128] Bianchini C., Frediani M., Giambastiani G., Kaminsky W., Meli A., Passaglia E. (2005). Amorphous Polyethylene by Tandem Action of Cobalt and Titanium Single-Site Catalysts. Macromol. Rapid Commun..

[129] Komon Z.J.A., Bu X., Bazan G.C. (2000). Synthesis of Butene−Ethylene and Hexene−Butene−Ethylene Copolymers from Ethylene via Tandem Action of Well-Defined Homogeneous Catalysts. J. Am. Chem. Soc..

[130] Komon Z.J.A., Diamond G.M., Leclerc M.K., Murphy V., Okazaki M., Bazan G.C. (2002). Triple Tandem Catalyst Mixtures for the Synthesis of Polyethylenes with Varying Structures. J. Am. Chem. Soc..

[131] Karbach F.F., Macko T., Duchateau R. (2016). Preparation of Ethylene/1-Hexene Copolymers from Ethylene Using a Fully Silica-Supported Tandem Catalyst System. Macromolecules.

[132] Markel E.J., Weng W., Peacock A.J., Dekmezian A.H. (2000). Metallocene-Based Branch−Block Thermoplastic Elastomers. Macromolecules.

[133] Liu S., Motta A., Delferro M., Marks T.J. (2013). Synthesis, Characterization, and Heterobimetallic Cooperation in a Titanium–Chromium Catalyst for Highly Branched Polyethylenes. J. Am. Chem. Soc..

[134] Liu S., Motta A., Mouat A.R., Delferro M., Marks T.J. (2014). Very Large Cooperative Effects in Heterobimetallic Titanium-Chromium Catalysts for Ethylene Polymerization/Copolymerization. J. Am. Chem. Soc..

[135] Arriola D.J., Carnahan E.M., Hustad P.D., Kuhlman R.L., Wenzel T.T. (2006). Catalytic Production of Olefin Block Copolymers via Chain Shuttling Polymerization. Science.

[136] Hustad P.D., Kuhlman R.L., Arriola D.J., Carnahan E.M., Wenzel T.T. (2007). Continuous Production of Ethylene-Based Diblock Copolymers Using Coordinative Chain Transfer Polymerization. Macromolecules.

[137] Hustad P.D. (2009). Frontiers in Olefin Polymerization: Reinventing the World’s Most Common Synthetic Polymers. Science.

[138] Hustad P.D., Marchand G.R., Garcia-Meitin E.I., Roberts P.L., Weinhold J.D. (2009). Photonic Polyethylene from Self-Assembled Mesophases of Polydisperse Olefin Block Copolymers. Macromolecules.

[139] Arora A., Fosfuri A., Gambardella A. (2004). Markets for Technology: The Economics of Innovation and Corporate Strategy.

[140] Chaudhary B.I., Barry R.P. (1999). Extruded Non-Crosslinked Foams Made from Ethylene-Styrene Interpolymers and Blends with Polyethylene. J. Cell. Plast..

[141] Timmers F.J. (1997). Pseudo-Random Copolymers Formed by Use of Constrained Geometry Addition Polymerization Catalysts. U.S. Patent.

[142] Chum P.S., Kruper W.J., Guest M.J. (2000). Materials Properties Derived from INSITE Metallocene Catalysts. Adv. Mater..

[143] Thayer A.M. (1995). Metallocene Catalysts Initiate New Era in Polymer Synthesis. Chem. Eng. News Arch..

[144] Severn J.R., Chadwick J.C., Duchateau R., Friederichs N. (2005). “Bound but Not Gagged”Immobilizing Single-Site α-Olefin Polymerization Catalysts. Chem. Rev..

[145] Severn J.R., Chadwick J.C. (2013). Immobilisation of homogeneous olefin polymerisation catalysts. Factors influencing activity and stability. Dalton Trans..

[146] Busico V. (2007). Catalytic Olefin Polymerization is a Mature Field. Isn’t it?. Macromol. Chem. Phys..

